# Supplementary material for: Spin-orbit interaction of light induced by transverse spin angular momentum engineering
Source: Nat Commun. 2018 Mar 2;9:926. doi: 10.1038/s41467-018-03237-5 (PMC5834641; doi:10.1038/s41467-018-03237-5)
Supplement: Supplementary file 1 — Supplementary Information [file 41467_2018_3237_MOESM1_ESM.pdf]

Spin-orbit interaction of light induced by transverse spin angular momentum engineering

by Shao et al.

Spin-orbit interaction of light induced by transverse spin angular momentum engineering

Zengkai Shao[1†], Jiangbo Zhu[2†], Yujie Chen[1], Yanfeng Zhang[1*], and Siyuan Yu[1,2*]

[1]School of Electronics and Information Engineering, State Key Laboratory of Optoelectronic Materials and Technologies, Sun Yat-sen University, Guangzhou 510275, China.
[2]Photonics Group, School of Computer Science, Electrical and Electronic Engineering, and Engineering Maths, University of Bristol, Bristol BS8 1UB, UK.
[†]These authors contributed equally to this work. [*]Correspondence and requests for materials should be addressed to Y. Zhang (email: zhangyf33@mail.sysu.edu.cn) or S. Yu (email: s.yu@bristol.ac.uk).

Supplementary Note 1. Scattering Effect of Grating Elements

The sub-micron particles used as 2nd-order grating in this study are of a square cuboidal shape (with dimensions $a\times a\times h$), and the orientation of their edges are parallel to the local coordinates (r, φ, z) of ring resonator, as shown in Supplementary Figure 1. The scatterers are of the same height as the waveguide (i.e., h = 0.4 μm or 0.6 μm in this paper), and attached to the inner-sidewall.

For scattering the virtually transverse-polarized [1] (i.e., in the x-y plane) light fields bounded in the sidewall evanescent region, it is essentially the transverse cross-sectional area (i.e., a^2) of such cuboids that dictates the scattering behaviour of the grating [2]. The transverse size of each grating element in the proof-of-concept devices here is a = 100 nm, considering the resolution limits of electron beam lithography used for defining the waveguide and gratings. The scattering of particles of such transverse size, given the wavelength of illumination λ = 1550 nm in this study, can be well described by the Rayleigh scattering approximation as $a << \lambda$ [3]. That is, the light scattered from each grating element can be approximated by radiation from a dipole antenna, with the phase locked to the driving transverse evanescent fields [3]. The polarizability of such cuboidal scatterers, α, is in general a tensor that quantifies the dipole moment **p** induced by external field **E** [3]

$$\mathbf{p} = \alpha\mathbf{E} \tag{1}$$

However, the fact that the sides of cuboidal scatterers are orientated along the axes of local coordinates (r, φ, z) results in a reduced form of polarizability as a diagonal matrix [2]

$$\alpha = \begin{bmatrix} \alpha_{rr} & 0 & 0 \\ 0 & \alpha_{\varphi\varphi} & 0 \\ 0 & 0 & \alpha_{zz} \end{bmatrix} \tag{2}$$

where α_{ii} ($i = r, \varphi, z$) represents the polarizability for dipole moment component p_i with an external field E_i. Considering the z-component electric field is negligible at sidewalls, the local scattering of evanescent wave at each grating element can be described as

$$\begin{bmatrix} p_r \\ p_\varphi \\ 0 \end{bmatrix} = \begin{bmatrix} \alpha_{rr} & 0 & 0 \\ 0 & \alpha_{\varphi\varphi} & 0 \\ 0 & 0 & \alpha_{zz} \end{bmatrix} \begin{bmatrix} E_r \\ E_\varphi \\ 0 \end{bmatrix}, \text{ or, } \begin{cases} p_r = \alpha_{rr} E_r \\ p_\varphi = \alpha_{\varphi\varphi} E_\varphi \end{cases} \tag{3}$$

Note that under Rayleigh approximation α_{ii} ($i = r, \varphi$) is a real number [2], as here the absorption by SiN_x scatterers can be neglected (cf. [4]), and no instantaneous phase shift should be imparted on the scattered

fields. As a result, the two transverse electric components (i.e., E_r and E_φ) retain their $\pm\pi/2$ phase difference with each other after scattering. This assumption is justified by the Stokes polarimetry performed on the near-field emission of devices (shown in Figure 5 of main text), as the measured Stokes parameters as a function of the azimuthal position (φ) are all practically in-phase with the curves predicted by theory. In other words, any phase change induced by scattering of E_r and E_φ will alter their phase difference from $\pm\pi/2$, so that tangible phase shifts between the measured and theoretical Stokes parameter curves will have appeared here.

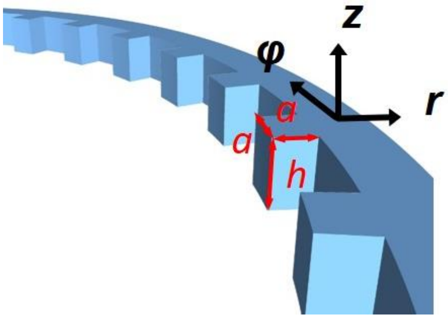

Supplementary Figure 1. Geometry of the Rayleigh scatterers placed in the evanescent region. All the scatterers used in this work are in a square cuboidal shape with the dimensions of $a\times a\times h$ (a = 100 nm for the fabricated devices and h is the waveguide height), and the edges of scatterers are aligned with the local coordinates (r, φ, z) to ensure no crosstalk among different field components is induced by scattering.

Supplementary Note 2. Phase-matching in CVV Scattering

The 2nd-order-grating-like scatterers distributed along the ring resonator perturb the evanescent wave of whispering-gallery modes (WGMs) circulating in the ring, and the scattered waves collectively produce vertically propagating waves. The interaction among the WGMs, grating, and scattered waves is generally governed by the following angular phase-matching condition

$$\mathbf{K}_{\text{WGM}} + \mathbf{K}_{\text{grating}} = \boldsymbol{\varphi} \cdot k_\varphi \tag{4}$$

where $\mathbf{K}_{\text{WGM}} = \pm\boldsymbol{\varphi}\cdot 2\pi n_{\text{eff}}/\lambda = \pm\boldsymbol{\varphi}\cdot p/R$ and $\mathbf{K}_{\text{grating}} = \mp\boldsymbol{\varphi}\cdot 2\pi/\Lambda = \mp\boldsymbol{\varphi}\cdot q/R$ are the local wave vectors of the WGM and of the angular grating, respectively (the opposite signs of these two vectors indicates they are always in the opposite angular directions, $\boldsymbol{\varphi}$ is the azimuthal unit vector, n_{eff} is the effective index of WGM, Λ is the period of grating, p is the azimuthal order of WGM, q is the number of grating elements, and R is the radius of resonator). $k_\varphi = \boldsymbol{\varphi}\cdot\mathbf{K}_{\text{sca}}$ denotes the φ-component of the scattered wave vector, as illustrated in Supplementary Figure 2. As a result, the angular phase-matching can also be written as

$$k_\varphi = \frac{\pm(p-q)}{R} \tag{5}$$

Here we define the azimuthal propagation constant (phase shift per unit azimuthal angle) of the scattered wave as $l = k_\varphi/R$, or, $l = \pm(p - q)$. Note here the choice of the sign (±) depends on the direction of WGM (i.e., clockwise or counter-clockwise). The scattered vector wave thus possesses a helical wavefront as

$$\mathbf{E}_{\text{sca}} \propto \left(\mathbf{r}\cdot E_r + \boldsymbol{\varphi}\cdot E_\varphi\right)\exp\left(il\varphi\right) \tag{6}$$

and non-zero orbital angular momentum (OAM) can be observed if the system is configured away from the Bragg reflection condition, i.e., $p - q \neq 0$.

To sum up, the physical interpretation of the helical wavefront in CVVs can be made as follows: the WGM circulating around the resonator carries a spatial phase profile as $\exp(\pm ip\varphi)$ (i.e., represented by the wave vector $\mathbf{K}_{\text{WGM}}$), is scattered by the angular 2nd-order grating and imparted with a 1st-order-diffraction phase as $\exp(\mp iq\varphi)$ (i.e., characterized by the vector $\mathbf{K}_{\text{grating}}$). And therefore, the scattered wave exhibits a spatial phase as $\exp(il\varphi)$ with a topological charge $l = \pm(p - q)$.

The above phase-matching rule is elaborated in [5].

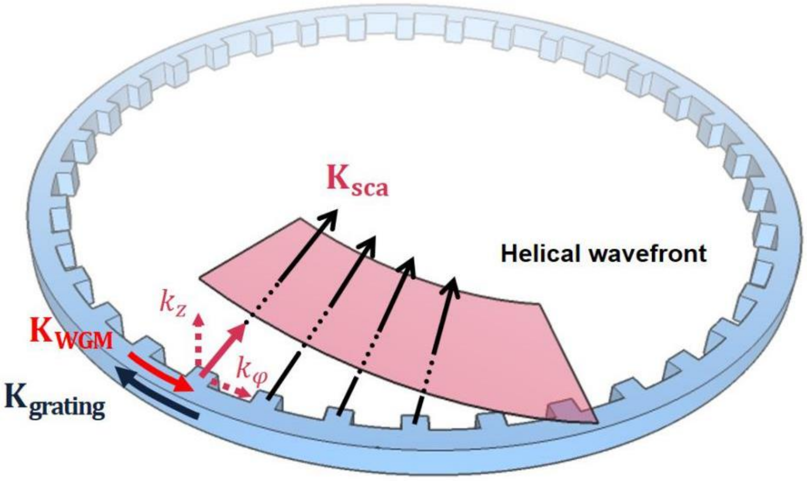

Supplementary Figure 2. Illustration of angular phase-matching in the emission of cylindrical vector vortex beams from the angular grating based devices. The deviation from the Bragg reflection in the ring resonator (i.e., the mismatch between the azimuthal mode order of WGM and the grating element number) gives rise to the helical wavefront and non-zero OAM carried by the scattered waves.

Supplementary Note 3. Transverse Spin State in Evanescent Wave

The transverse spin state in the perturbed evanescent wave is defined as

$$\sigma = \frac{|E_{\text{L}}|^2 - |E_{\text{R}}|^2}{|E_{\text{L}}|^2 + |E_{\text{R}}|^2} \tag{7}$$

where E_{L} and E_{R} are the local left- and right-hand circular polarization components in the evanescent wave, respectively, under the cylindrical basis (r, φ)

$$E_{\mathrm{L}} = \frac{1}{\sqrt{2}}\begin{bmatrix}1\\i\end{bmatrix}^{\dagger}\mathbf{E}_{\mathrm{eva}} = \frac{1}{\sqrt{2}}\left(E_{rr} - iE_{\varphi\varphi}\right) \quad (8)$$

$$E_{\mathrm{R}} = \frac{1}{\sqrt{2}}\begin{bmatrix}1\\-i\end{bmatrix}^{\dagger}\mathbf{E}_{\mathrm{eva}} = \frac{1}{\sqrt{2}}\left(E_{rr} + iE_{\varphi\varphi}\right) \quad (9)$$

in which $[*]^{\dagger}$ denotes the conjugate transpose of the right- or left-hand circular polarization Jones vector, and $\mathbf{E}_{\mathrm{eva}} \propto [E_{rr}\ E_{\varphi\varphi}]^{\mathrm{T}}$ ($E_{rr} = W_{rr}E_r$ and $E_{\varphi\varphi} = W_{\varphi\varphi}E_{\varphi}$). As a result, the transverse spin state can be explicitly rewritten as

$$\sigma = \frac{i\left(E_{rr}E_{\varphi\varphi}^{*} - E_{rr}^{*}E_{\varphi\varphi}\right)}{\left|E_{rr}\right|^{2} + \left|E_{\varphi\varphi}\right|^{2}} \quad (10)$$

Additionally, note that E_{rr} and $E_{\varphi\varphi}$ retain their $\pm\pi/2$ phase difference after grating scattering (Supplementary Note 1), and thus the local polarization unit vector in the polar basis always follows the form as $[E_{rr}\ E_{\varphi\varphi}]^{\mathrm{T}} = [\cos\gamma\ \pm i\sin\gamma]^{\mathrm{T}}$, where γ is a real number. If we denote here the ratio of the two components by a real number $\kappa = iE_{\varphi\varphi}/E_{rr}$, the dependence of σ on this ratio can be obtained as

$$\sigma = -\frac{2\kappa}{1+\kappa^{2}} \quad (11)$$

Supplementary Note 4. Formulation of Cylindrical Vector Vortices Emission

The phase-matching condition that governs the WGM and grating scatterers has been discussed in the previous Note to show the essence of this device. However, to fully present the interaction between the transverse spin in evanescent waves and the OAM in CVVs, a more explicit formulation is presented here. The evanescent wave of WGMs, written in the cylindrical polarization basis as $\mathbf{E}_{\mathrm{in}} \propto e^{\pm ip\varphi}[E_r\ E_{\varphi}]^{\mathrm{T}}$, is perturbed by the second-order-grating-like scatterers when circulating around the resonator. Here we denote the positive integer p as the azimuthal mode number of WGMs, and the two counter-propagating degenerate WGMs resonating in the same wavelength have the mode numbers of p (counter-clockwise, CCW) and $-p$ (clockwise, CW), respectively. The perturbation of gratings to the evanescent wave is generalized in a matrix as

$$\mathbf{M}_1 = \begin{bmatrix}W_{rr} & 0\\0 & W_{\varphi\varphi}\end{bmatrix}\cdot e^{i\delta(\varphi)} \quad (12)$$

where W_{rr} and $W_{\varphi\varphi}$ are real numbers that reflect the modulation on the amplitudes of local transverse (E_r) and longitudinal (E_{φ}) fields, respectively, due to grating perturbation (see Supplementary Note 1). The off-diagonal elements of $\mathbf{M}_1$ are vanishing as we assume the scattering does not introduce coupling between orthogonal field components (Supplementary Note 1). Note that the phase acquired in scattering $\delta(\varphi)$ is not an instantaneous phase shift to the scattered fields, but only represents the relative

phase delay between the scattered waves at different locations. From the phase-matching condition above (i.e., $l = p - q$) and the spatial phase of WGMs (i.e., $\pm p\varphi$), it's straightforward to find that $\delta(\varphi) = \mp q\varphi$. This result agrees with the phase imparted on the first-order wave in 2nd-order-grating diffraction derived using coupled-mode theory (cf. supplementary material of ref. [5]).

In addition, as WGMs travel around the resonator, the vector evanescent wave experiences a rotation of local coordinates (r, φ) with respect to the global laboratory frame (x, y), as shown in main text Figure 1c. The effect of this rotation on the emitted CVVs (represented in the basis of $[E_x\ E_y]^{\mathrm{T}}$) can be written with a single matrix $\mathbf{M}_2$ as

$$\begin{bmatrix} E_x \\ E_y \end{bmatrix} = \mathbf{M}_2 \cdot \begin{bmatrix} E_{rr} \\ E_{\varphi\varphi} \end{bmatrix} = \begin{bmatrix} \cos\varphi & \mp\sin\varphi \\ \pm\sin\varphi & \cos\varphi \end{bmatrix} \begin{bmatrix} E_{rr} \\ E_{\varphi\varphi} \end{bmatrix} \tag{13}$$

The final output CVV ($\mathbf{E}_{\text{out}} = \mathbf{M}_2 \cdot \mathbf{M}_1 \cdot \mathbf{E}_{\text{in}}$) can be obtained in the basis of circular polarizations

$$\mathbf{E}_{\text{out}} \propto \frac{1}{2}\left\{ e^{i(l_{\text{TC}}-1)\varphi}\left(E_{rr} - iE_{\varphi\varphi}\right)\begin{bmatrix} 1 \\ i \end{bmatrix} + e^{i(l_{\text{TC}}+1)\varphi}\left(E_{rr} + iE_{\varphi\varphi}\right)\begin{bmatrix} 1 \\ -i \end{bmatrix} \right\} \tag{14}$$

where $l_{\text{TC}} = \pm(p - q)$ is the topological charge. By incorporating the transverse spin state σ defined in Supplementary Equation (10), the output can be written more explicitly as a function of σ

$$\mathbf{E}_{\text{out}} \propto \frac{1}{2}\left\{ \sqrt{1+\sigma}\, e^{i(l_{\text{TC}}-1)\varphi}\begin{bmatrix} 1 \\ i \end{bmatrix} + \sqrt{1-\sigma}\, e^{i(l_{\text{TC}}+1)\varphi}\begin{bmatrix} 1 \\ -i \end{bmatrix} \right\} \tag{15}$$

Supplementary Note 5. Angular Momentum in Cylindrical Vector Vortices

The cylindrical vector vortices (CVVs) emitted from the angular-grating based devices considered in this paper exhibit good paraxiality, as the radius of ring resonator (R = 80 μm) is much larger than the wavelength (λ = 1.55 um) [6, 7]. The angular momentum (AM) carried in paraxial optical vortex beams can be essentially considered as the sum of the spin and orbital AM components, which are associated with the polarization and spatial properties of light, respectively [8, 9]. The cycle averaged z-component of the spin AM (SAM) and orbital AM (OAM) per unit length per photon of a vortex beam can be written as [9]

$$S_z = \frac{\hbar \iint r dr d\varphi \left(E_x^* E_y - E_y^* E_x\right)}{i \iint r dr d\varphi\, \mathbf{E}^* \cdot \mathbf{E}} \tag{16}$$

$$L_z = \frac{\hbar \iint r dr d\varphi \sum_{j=x,y,z} E_j^* \frac{\partial}{\partial\varphi} E_j}{i \iint r dr d\varphi\, \mathbf{E}^* \cdot \mathbf{E}} \tag{17}$$

By substituting the CVV shown in Supplementary Equation (15) into the equations above, the SAM and OAM components carried by the CVV are

$$S_z = \sigma\hbar \tag{18}$$

$$L_z = \left(l_{\mathrm{TC}} - \sigma\right)\hbar \tag{19}$$

where σ is the transverse-spin state in the near-field evanescent wave. The total angular momentum (TAM) in a CVV ($J_z = S_z + L_z$) is thus simply written as

$$J_z = l_{\mathrm{TC}}\hbar \tag{20}$$

Supplementary Note 6. Geometric Phase Induced by Coordinate Rotation

As the polarization state of CVVs is space-variant [6], here the Pancharatnam phase is used to define the phase difference of light fields in different positions in CVVs [7], that $\Phi_{\mathrm{P}} = \arg\langle \mathbf{E}(r_1, \varphi_1), \mathbf{E}(r_2, \varphi_2)\rangle$, where $\arg\langle \mathbf{E}_1, \mathbf{E}_2\rangle$ is the argument of the inner product of the two Jones vectors $\mathbf{E}_1$ and $\mathbf{E}_2$. Following this definition, the Pancharatnam phase of fields at two different positions (r_1, φ_1) and (r_2, φ_2) in a CVV is given by

$$\Phi_{\mathrm{P}} = l_{\mathrm{TC}}\Delta\varphi + \arg\left(\cos\Delta\varphi \mp i\sigma\sin\Delta\varphi\right) \tag{21}$$

where $\Delta\varphi = \varphi_2 - \varphi_1$, and the CVV excited by CCW (CW) WGM takes the + (–) sign in the equation. The gradient of Pancharatnam phase along the azimuthal direction is

$$\lim_{\Delta\varphi\to 0}\frac{\Phi_{\mathrm{P}}}{\Delta\varphi} = l_{\mathrm{TC}} \mp \lim_{\Delta\varphi\to 0}\frac{\arctan\left(\sigma\tan\Delta\varphi\right)}{\Delta\varphi} = l_{\mathrm{TC}} \mp \sigma \tag{22}$$

Clearly, the Pancharatnam phase in CVVs is a linear function of coordinate φ, and thus we can rewrite it as

$$\Phi_{\mathrm{P}} = l_{\mathrm{TC}}\varphi \mp \sigma\varphi \tag{23}$$

Considering the SAM component carried by CVVs shown in Supplementary Equation (18), the Pancharatnam phase can be generalized as

$$\Phi_{\mathrm{P}} = l_{\mathrm{TC}}\varphi - \int \mathbf{S}\cdot\mathbf{\Omega}_{\varphi}d\varphi \tag{24}$$

where $\mathbf{S} = S_z\hbar^{-1}\cdot\mathbf{z}$ is the SAM per photon, and $\mathbf{\Omega}_{\varphi}$ is the angular velocity of reference frame rotation with respect to the coordinate φ for Pancharatnam phase comparison (see main text Figure 1c). Here, $\mathbf{\Omega}_{\varphi} = \pm\mathbf{z}$ for CCW and CW WGMs, respectively.

Supplementary Note 7. Techniques for Polarization and OAM States Characterization

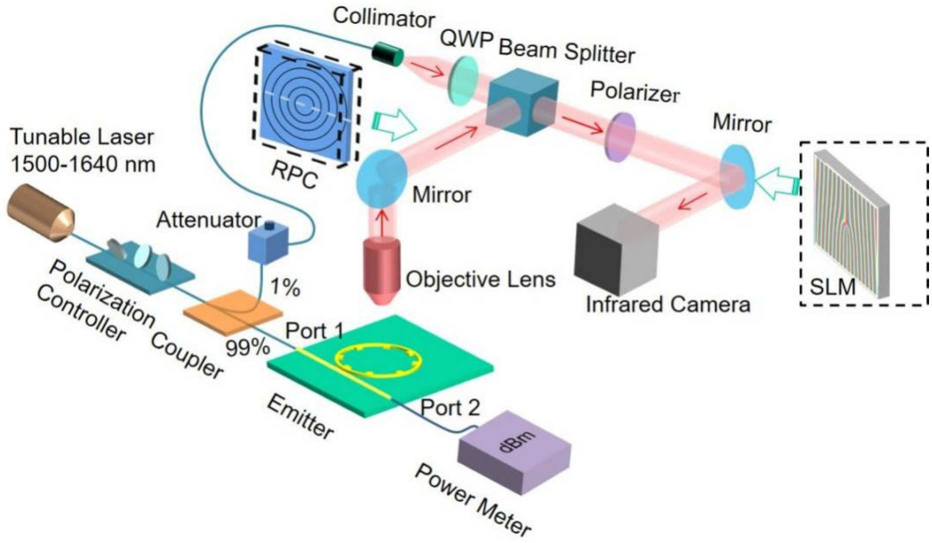

Supplementary Figure 3. Experimental setup for device characterization and the observation of the transverse-spin induced SOI effect.

The experimental characterizations of the devices are performed with the setup shown in Supplementary Figure 3. For the excitation of WGMs and hence emission of CVVs, the continuous-wave light from the tunable laser source (8461B, Agilent) is controlled with a fiber polarization controller (FPC561, Thorlabs), and the quasi-TE mode in the waveguide is excited by launching the horizontally polarized light into one of the ports (e.g., Port 1 as shown in Supplementary Figure 3) using a lensed fiber (SMF-28E+LL, Corning). A small fraction, 1%, of the input light is tapped using a coupler (PMC1550-90B-FC, Thorlabs) and directed to another collimator (F240FC-1550, Thorlabs) to serve as the reference light for the interference with the emitted CVVs.

For the measurement of the emission spectrum of the device, the vertically emitted beam from the device plane is collected and collimated with a 20X objective lens (UPlanFLN, Olympus) positioned in the working distance (1.7mm) away from the device. A power meter (PM122D, Thorlabs) is placed behind the collimating objective lens to record the dependence of emission power on the working wavelength, while the output wavelength of the tunable laser is swept from 1500 nm to 1640 nm with the step of 10 pm.

For measuring the average cylindrical-basis polarization ellipticity of CVVs, a liquid crystal based element called Radial Polarization Converter (RPC, ARCoptix S. A., Switzerland) is used to selectively measure the power of E_φ and E_r components. The RPC can be typically used for its spatially varying anisotropy to convert linearly polarized light into vector beams of azimuthal or radial polarizations [11]. Here the reversed effect of this element is employed: by injecting the light into the exit side, E_φ and E_r in the CVV will be converted into x- and y-polarized light leaving the entrance side, respectively. A linear polarizer (LPNIR100-MP2, Thorlabs) is then used to filter out one of the

components, and by detecting the power of the two orthogonal components as P_φ and P_r, the squared polarization ellipticity (ε^2) in the CVV determined by the near-field transverse spin state can be obtained as $\varepsilon^2 = P_r/P_\varphi$ or P_φ/P_r.

For Stokes parameters measurements, the near-field pattern of the CVV is imaged onto an InGaAs camera (C14041-10U, Hamamastu) with an achromatic lens (f = 250 mm, AC254-250-C-ML, Thorlabs), and the linear- and circular-polarizations are obtained by adjusting the quarter-wave plate (QWP, AQWP 10M-1600, Thorlabs) and the linear polarizer (LP) mounted on continuous rotation mounts (CRM1, Thorlabs).

For the characterization of OAM states in CVVs, a phase-only reflective spatial light modulator (PLUTO SLM, HOLOEYE Photonics AG) loaded with grey-scale fork-grating patterns is used [12]. A linear polarizer is first used to acquire one of the linear-polarized components in the CVV, which generally is a mixture of two topologically charged vortices as shown in Equation (4) in the main text. The central axis of the polarized CVVs is then aligned with the center of fork-grating patterns on the SLM. For each incident CVV, the SLM is loaded with a series of fork-grating images with consecutive integer topological charges, e.g., l_{SLM} = -5, -4, …, +5. The light reflected off each image is focused by an achromatic lens (f = 150 mm, AC254-150-C-ML, Thorlabs) followed by the InGaAs camera, and the power of the corresponding OAM component l_{SLM} is obtained by integrating the intensity of the central Gaussian-like spot [13]. The process is repeated for the other linear-polarized component, and the measured OAM spectrum of the incident CVV is then obtained by averaging the two corresponding OAM components over the two linear polarization components.

Supplementary Note 8. Preliminary Characterization of Devices

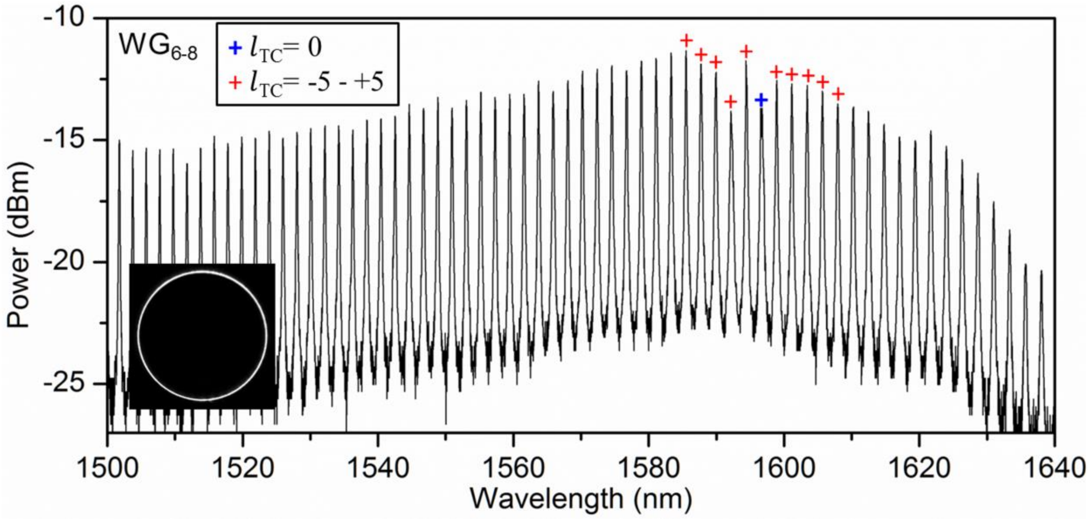

Supplementary Figure 4. Measured emission spectral response of sample device $W_{6\text{-}8}$ as input wavelength is swept from 1500-1640 nm. The inset shows a typical near-field intensity profile of emitted CVVs.

The measured emission spectral response of sample $WG_{6\text{-}8}$, as an instance, is plotted in Supplementary Figure 4 after normalization to the output power of tunable laser. The central wavelength at which the emitted CVV has $l_{TC} = p - q = 0$, is $\lambda_c = 1596.6$ nm, and the free spectral range is around 2.2 nm. At the wavelengths longer (shorter) than λ_c, CVVs carry positive (negative) integer l_{TC} at the resonance peaks. The inset shows a typical near-field intensity profile of the device at the resonance wavelengths. The long-range variation of peak emitted power across the spectral range is primarily caused by the fixed gap between access waveguide and ring resonator that couples varying power into the resonator across the spectrum.

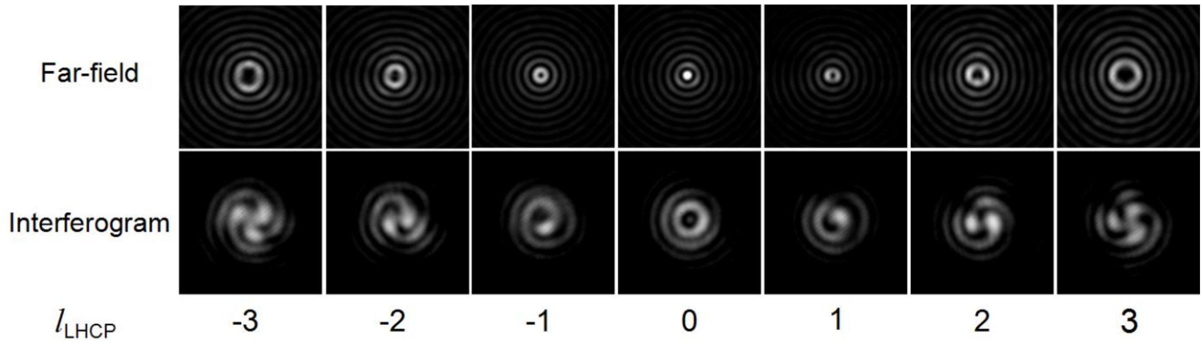

Supplementary Figure 5. Far-field profiles and interferograms of left-hand circular-polarized components of CVVs from device $WG_{6\text{-}8}$.

Some typical far-field intensity profiles and interferograms of CVVs are illustrated in Supplementary Figure 5, in which the device $WG_{6\text{-}8}$ is configured for the emission of CVVs with l_{TC} from –2 to +4. The left-hand circular polarized (LHCP) component is obtained by filtering the far-field CVVs with a QWP and LP combination, and then interferes with the LHCP Gaussian beam. For each CVV of l_{TC}, the LHCP component possesses the OAM state of $l_{LHCP} = l_{TC} - 1$ (see Equation (4) in the main text), and therefore each interferogram shown in the figure clearly exhibits the spiral fringes with the number of l_{LHCP} [5].

Supplementary Note 9. Discussion on the Measured Stokes Parameters

Generally, the fluctuations in the measured Stokes parameters along the resonator (azimuthal direction), as shown in Figure 5 in the main text, are attributed to the non-uniformity of fabricated gratings, as well as the decaying intensity of WGMs along the resonator. The deviation of measurements from the theory is more evident with devices of smaller $|S_3|$. This is possibly caused by the light that is scattered from the other (outer-) side of waveguide, carrying the opposite σ, due to sidewall roughness. In some devices, standing-wave-like patterns (e.g., map (iv) in Figure 5c) are introduced by the interference of scattered co-existing TE and TM modes, because in these waveguide designs these two

modes are more degenerate and single-polarization-mode excitation is more critical to polarization control in mode launching.

Supplementary Note 10. Experimental Setup for Spin-Orbit Unidirectional Coupling

The experimental setup for the measurement of spin-orbit controlled unidirectional coupling is shown in Supplementary Figure 6. The polarized light from the tunable laser is collimated with a collimator and then reflected by the SLM for the conversion to the vortex carrying OAM state l_{in}. The linear-polarized vortex is imparted a certain polarization state (σ_{in}) by the rotatable QWP. A 20X objective lens is used for focusing and illuminating the prepared vortex of spin and orbital AM states $<\sigma_{in}, l_{in}>$ onto the device. Two lensed fibers are used for collecting the received power from the waveguide Ports 1 and 2, respectively.

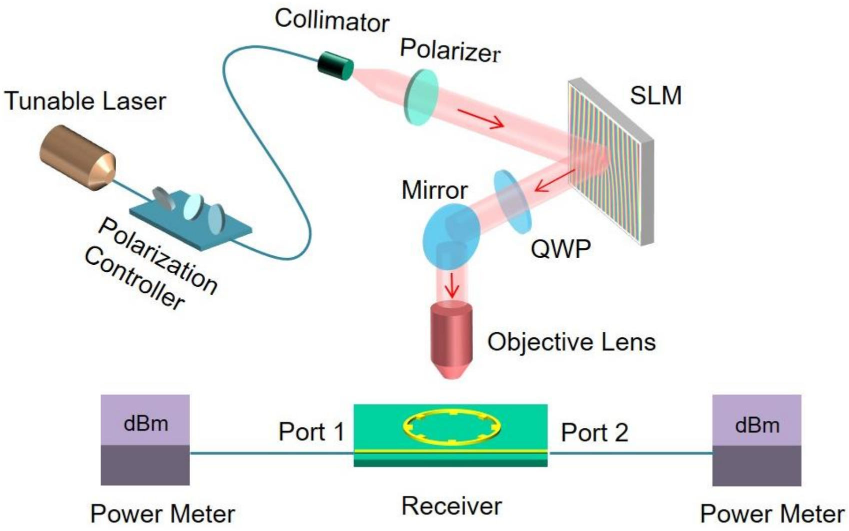

Supplementary Figure 6. Experimental setup for the measurement of spin-orbit controlled directional coupling of waveguide modes.

Supplementary References

[1] Marcuvitz, N. *Waveguide Handbook 1st edn* (McGraw-Hill, New York, 1951).

[2] Farafonov, V. G. & Il'in, V. B. Rayleigh approximation for light scattering at parallelepipeds. *J. Opt. Technol.* **81**, 375-381 (2014).

[3] Born, M. & Wolf, E. *Principles of Optics 6th edn* (Pergamon, Oxford, 1980).

[4] Luke, K., Okawachi, Y., Lamont, M. R. E., Gaeta, A. L. & Lipson, M. Broadband mid-infrared frequency comb generation in a Si3N4 microresonator. *Opt. Lett.* **40**, 4823-4826 (2015).

[5] Cai, X., Wang, J., Strain, M. J., Johnson-Morris, B., Zhu, J., Sorel, M., O'Brien, J. L., Thompson, M. G. & Yu, S. Integrated compact optical vortex beam emitters. *Science* **338**, 363-366 (2012).

[6] Zhu, J., Cai, X., Chen, Y. & Yu, S. Theoretical model for angular grating-based integrated optical vortex beam emitters. *Opt. Lett.* **38**, 1343-1345 (2013).

[7] Zhu, J., Chen, Y., Zhang, Y., Cai, X. & Yu, S. Spin and orbital angular momentum and their conversion in cylindrical vector vortices. *Opt. Lett.* **39**, 4435-4438 (2014).

[8] Allen, L., W. Beijersbergen, M., Spreeuw, R. J. C. & Woerdman, J. P. Orbital angular momentum of light and the transformation of Laguerre-Gaussian laser modes. *Phys. Rev. A* **45**, 8185-8189 (1992).

[9] Barnett, S. M. & Allen, L. Orbital angular momentum and nonparaxial light beams. *Opt. Commun.* **110**, 670-678 (1994).

[10] Pancharatnam, S. Generalized theory of interference and its applications. Part I. Coherent pencils. *Proc. Ind. Acad. Sci.* **44**, 247-262 (1956).

[11] Stalder, M. & Schadt, M. Linearly polarized light with axial symmetry generated by liquid-crystal polarization converters. *Opt. Lett.* **21**, 1948-1950 (1996).

[12] Gibson, G., Courtial, J., Padgett, M. J., Vasnetsov, M., Pas'ko, V., Barnett, S. M. & Franke-Arnold, S. Free-space information transfer using light beams carrying orbital angular momentum. *Opt. Express* **12**, 5448-5456 (2004).

[13] Strain, M. J., Cai, X., Wang, J., Zhu, J., Phillips, D. B., Chen, L., Lopez-Garcia, M., O'Brien, J. L., Thompson, M. G., Sorel, M. & Yu, S. Fast electrical switching of orbital angular momentum modes using ultra-compact integrated vortex emitters. *Nat. Commun.* **5**, 4856 (2014).
